# Investigation of the uptake of molybdenum by plants from Argentinean groundwater

**DOI:** 10.1007/s11356-021-13902-w

**Published:** 2021-04-30

**Authors:** Kathryn Lawson-Wood, Maisarah Jaafar, Mónica Felipe-Sotelo, Neil I. Ward

**Affiliations:** 1 ICP-MS Facility, Department of Chemistry, University of Surrey, Guildford, Surrey GU2 7XH UK; 2 Present Address: Perkin Elmer, Chalfont Road, Seer Green, Buckinghamshire HP9 2FX UK; 3 Faculty of Science and Marine Environment, Universiti Malaysia Terengganu, 21030 Kuala Nerus, Terengganu Malaysia

**Keywords:** Arsenic, Atomic spectroscopy, Groundwater, Hydroponics, Molybdenum, Plants, Uptake, Vanadium

## Abstract

Some regions of Argentina are affected by high concentrations of molybdenum, arsenic and vanadium from natural sources in their groundwater. In particular, Mo levels in groundwater from Eduardo Castex (La Pampa, Argentina) typically exceed the guidelines for drinking water formerly established by WHO at 70 μg/L. Therefore, this study investigated the uptake of Mo in plants, using cress (*Lepidium sativum* L*.*) as a model using hydroponic experiments with synthetic solutions and groundwater from La Pampa. Cress grown from control experiments (150 μg/L Mo, pH 7) presented an average Mo concentration of 35.2 mg/kg (dry weight, d.w.), higher than the typical total plant range (0.7–2.5 mg/kg d.w.) in the literature. Using pooled groundwater samples (65.0–92.5 μg/L Mo) from wells of La Pampa (Argentina) as growth solutions resulted in significantly lower cress Mo levels (1.89–4.59 mg/kg d.w.) than were obtained for synthetic solutions of equivalent Mo concentration. This may be due to the high levels in these groundwater samples of As, V, Fe and Mn which are known to be associated with volcanic deposits. This research addressed the hitherto scarcity of data about the effect of various physicochemical parameters on the uptake of Mo in plants.

**Supplementary Information:**

The online version contains supplementary material available at 10.1007/s11356-021-13902-w.

## Introduction

Relatively high levels of molybdenum have been observed in groundwater under oxic or alkaline conditions associated with some volcanic terrains. Under these conditions, high levels of Mo (2.7–90 μg/L) have been reported in groundwater affected by rhyolitic ash deposits of the Chaco-Pampean plain, Argentina (Smedley and Nicolli 2014). Although the former guideline limit for Mo in drinking water established at 70 μg/L has been withdrawn by WHO (2011), levels over that limit have been reported in some regions of the world, including Argentina. Therefore, it has been recommended that individual stakeholders should establish their own limits for Mo in drinking water, or at least continue using the former WHO guidelines set at 70 μg/L (Frisbie et al. 2015). Moreover, elevated levels of arsenic O’Reilly et al. 2010; Watts et al. 2010; Jaafar et al. 2018) and vanadium (Al-Rawahi and Ward 2017) have also been found in surface and groundwater of Argentina, including the province of La Pampa.

In biological systems, Mo is an essential constituent (pterin cofactor) of various enzymes, collectively known as molybdoenzymes, which catalyse key reduction–oxidation reactions. Nitrate reductase and nitrogenase are two major molybdoenzymes involved in nitrogen metabolism in plants (Tejada-Jiménez et al. 2013). The requirements of Mo in many plants are, in general, extremely low in comparison with other micronutrients (including manganese and chloride) and it is rather unusual to find a Mo deficiency condition in plants (Broadley et al. 2012). Plants are generally resistant to high Mo concentrations. However, Mo toxicity can be exhibited by yellowing of leaves and depressed root growth (McGrath et al*.* 2010). While information regarding plant Mo toxicity is limited, there are reports of toxic levels (135 mg/kg Mo (d.w.)) in leaves and shoots of young spring barley (Davis et al. 1978).

Despite the reported high levels of Mo and other toxic elements (especially As) in farm groundwater (O’Reilly et al. 2010; Jaafar 2018), many rural regions in La Pampa (Argentina) have limited or no access to treated water. As such, they rely heavily on groundwater wells for the watering of livestock and for the irrigation of pastures and cultivars. Therefore, it is necessary to investigate for the first time the uptake of Mo (and other elements) present in these groundwater samples by plants, as this could constitute a route for the accumulation of Mo in foodstuffs used by animals and humans in regions like La Pampa, Argentina.

The uptake of Mo in plants was investigated by a series of hydroponic experiments using cress (*Lepidium sativum* L.) as the model cultivar, grown in synthetic solutions (tap water) and pooled groundwater samples collected from farms near Eduardo Castex (La Pampa, Argentina). This experimental approach has been widely used in plant nutrition research since the composition of chemicals in the hydroponic solutions and the growing conditions can be easily controlled. The elemental composition of all solutions was determined using atomic spectroscopy. Additional experiments were carried out using synthetic solutions with known spiked levels of Mo (150 μg/L) and added manganese, iron and aluminium (at concentrations typically found in Argentine groundwater from La Pampa), to determine the potential competitive or synergetic effects of other concomitant elemental species on the uptake of Mo by cress grown under hydroponic conditions. A further set of experiments was completed to assess the effect of added nutrients (using a commercial plant feed) as would be expected to happen in farms in La Pampa where such nutrients would come from the loess deposits, soil layer solutions and through farming practices (surface flooding and sprinkler irrigation).

## Materials and methods

### Collection of water samples

Groundwater samples were obtained from rural farm wells near Eduardo Castex (35°53′60S, 64°17′60W), La Pampa, Argentina and two composite samples (GW1 and GW2) were collected for the hydroponic experiments. Oral permission was given by the owners of each well and the field work was conducted in collaboration with Administración Provincial del Agua (APA, Santa Rosa, Provincia de La Pampa, Argentina) and Cospec LTDA (Eduardo Castex, Argentina). Groundwater was pumped using a fan-driven well head and the samples were drawn from the flow into a disposable, clean and rinsed (three times with the collected sample) 20-mL BD plastic syringe (BD Plastipak, Oxford, UK), filtered through a 0.45-μm syringe-driven unit filter (Millex-GP; Millipore, Hertfordshire, UK) into polypropylene bottles (Sterilin, Newport, UK) ready for transport. The composite groundwater samples (GW1 and GW2) for the hydroponic experiments were prepared by combination of the filtered solutions. The physicochemical parameters for the groundwater samples, namely, pH, electrical conductivity (EC—μS/cm), total dissolved solids (TDS—parts per million, ppm) and redox potential (E_h_—mV) were recorded using a calibrated Hanna HI 98129 Digital Combo Meter and a calibrated Hanna HI 98120 Digital ORP Meter (both from Hanna Instruments Ltd, Bedfordshire, UK). The data obtained for the two pooled groundwater samples (GW1 and GW2) were EC, 3386 and >3999 μS/cm; TDS, 1966 and >1999 ppm; and E_h_, 0.24 and 0.27 mV. The samples were stored at <4 °C until further analysis and use in the hydroponic experiments.

### Hydroponic experiments

Hydroponic experiments were carried out to assess the uptake of Mo in plants using control (tap water), spiked solutions (Mo with or without Fe, Mn and Al) and two pooled groundwater samples (GW1 and GW2) collected from farm wells in Eduardo Castex (La Pampa, Argentina). This experimental approach was selected as it allows for the simple control of the growing conditions. Cress seeds (fine curled cress, *L. sativum*, commercially available Mr. Fothergill’s Seeds, UK) were used in the hydroponic experiments as purchased, without any further pre-treatment. The seeds (50 per experiment replicate) were placed onto sections of polyester netting (400 cm^2^, 100% polyester, 1 mm mesh) and paper tissue, which had been pre-soaked in the appropriate growing solutions. These support materials were secured in place using elastic bands on top of a polypropylene cup (350 mL total capacity), filled with the solutions required for each hydroponic experiment (approximately 200 mL). The containers were kept in a ‘trace element free’ fume-hood fitted with two hydroponic lights (Toplanet 45W LED plant grow panel light—Red Blue White Plant Lamp), placed at a height of approximately 0.6 m from the top of the samples. The plants were grown under room temperature conditions, with oscillations of the temperature between 20 and 25 °C between night and day covering a photoperiod of 16/8 h. During the first few days of germination, the study solutions (control, spiked tap water and Argentine groundwater) were added to the respective containers every morning and evening, to ensure the level of water was kept at the height of the netting to maintain the tissue moisture and keep the roots in the growing solution. Thereafter, the plants were watered daily until the 12-day growth period was complete. Each hydroponic experiment was conducted in duplicate. Control samples were prepared using local tap water with pH 7.20, EC 120 μS/cm, TDS 62 ppm and E_h_ 23 mV. The effect of the pH on the Mo uptake was done by modifying the pH of each solution using ammonium hydroxide and hydrochloric acid (both from Fisher Scientific, Loughborough, UK), as required. The elemental concentrations of the control tap water (mean ± SD, *n*=3) were <0.1 μg/L (limit of detection, LOD) Mo, 0.20 ± 0.02 μg/L As, 0.14 ± 0.01μg/L V, 0.30 ± 0.02 μg/L Mn, 4.7 ± 0.1 μg/L Fe, 227 ± 1 μg/L Cu, 26.4 ± 0.2 μg/L Zn and 1.6 ± 0.1 μg/L Al, as determined by inductively coupled plasma mass spectrometry (ICP-MS; Agilent 7700x series).

The effect of concomitant ions in the water on plant Mo uptake was investigated through hydroponic experiments using control tap water spiked with known amounts of Mo, alone or in combination with Fe, Mn and Al, at similar levels to those found in the groundwater samples from La Pampa. Furthermore, under natural growth conditions, plants have other nutrients (N, P, K, Ca, Mg, etc) provided by the soil solution. Therefore, a hydroponic experiment was undertaken adding a commercial nutrient growth solution (Baby Bio, Bayern; NPK composition 10.6-1.9-1.4, % of N, P and K, respectively, in the concentrated product). This was used to spike the tap water at a level recommended for plants, 0.01% (v/v). Mo, Fe, Mn and Al stock solutions were prepared from analytical-grade standards (SPC Science, Courtaboeuf, France). At the pH–Eh conditions observed for the groundwater at the time of collection (see Collection of water samples section), the expected dominant species of Mo is MoO_4_^2−^ or HMoO_4_^−^, although at extremely low pH levels significant amounts of Mo^3+^ may also be present (Ochs et al. 2011).

After the 12-day growth period, the experimental plants were harvested by carefully removing the plant material from the tissue and netting. Shoots and roots were washed several times with doubly distilled water (DDW, 18 mΩ/cm) to remove remnants of solution that had not been absorbed by the plants. For individual growing containers, each plant was separated from one another, any non-germinated seeds were removed and 15 plants were selected at random from each container. For each of the selected 15 plants, the root and shoot lengths were measured to evaluate any effects on plant growth due to the composition of the experimental solution.

### Analytical procedures

#### Digestion of plant samples

For the elemental analysis of the plant material, the samples washed in DDW were dried in an oven for 24 h at 60 °C. Portions of each finely cut plant sample (0.25 ± 0.01 g, as d.w.) were placed into pre-weighed ceramic crucibles and dry ashed in a muffle furnace (Carbolite, UK) at 500 °C for 12 h. The ash samples were homogenised in a fume cupboard, and approximately 1 mL of concentrated HNO_3_ (trace metal analysis grade; Fisher Scientific, Loughborough, UK) was slowly added to the ash in the crucible. The solutions resulting from the treatment of the mineral residue with the concentrated acid were then transferred to 25-mL Sterilin vials and diluted with DDW. The crucibles were repeatedly washed with DDW to ensure complete transfer of the solutions into the vials. Finally, the digested solutions were filtered using a syringe-top 0.45 μm membrane filter (Millex-GP; Millipore, Hertfordshire, UK) into a clean 25-mL Sterilin vial to remove any undissolved solids; ready for analysis by ICP-MS.

#### Elemental analysis by ICP-MS

Trace element levels of water or plant samples were determined using a 7700x series ICP-MS with MassHunter Workstation software and ASX-500 series auto-sampler (Agilent Technologies, Stockport, UK). The sampling interface consisted of a Ni-tipped sampling cone and 0.4 mm Ni skimmer cone. The quadrupole mass analyser was fitted with a third-generation octopole reaction system (ORS^3^; Agilent) for the elimination of polyatomic interferences; all ICP-MS analyses were carried out using the collision cell in both no gas and helium modes for the removal of ^40^Ar^39^K^16^O^+^ polyatomic interference on ^95^Mo (May and Wiedmeyer 1998). Typical operating conditions are found in Table S1 (in Supplementary Information).

Multi-element calibration standards were prepared by mass using 1% HNO_3_ (Fisher Scientific, Loughborough, UK) from commercially available 1000 mg/L stock standard solutions (SCP Science, Courtaboeuf, France) over the calibration range 1–3000 μg/L for the analysis of digested plants and waters. A 100-μg/L multi-element internal standard (IS) was introduced to the ICP-MS via a T-piece and mixed with the sample solution during sample uptake. Preparation of the IS was achieved by dilution of a 100 mg/L internal standard mix containing ^115^In (Agilent Technologies, Stockport, UK) in trace metal analysis grade 1% HNO_3_.

#### Quality assurance for elemental analysis

Two certified reference materials, namely Polish Virginia Tobacco Leaves INCT-PVTL-6 (Institute of Nuclear Chemistry and Technology, Poland) and NIST SRM 1640a—Trace Elements in Natural Waters (National Institute of Standards and Technology, Gaithersburg MA, USA), were used to evaluate the levels of accuracy and precision for both the sample preparation methods and instrumental analyses of the water and plant samples. Table 1 shows the calculated levels of accuracy, repeatability (*n* = 6) and reproducibility (*n* = 3) data for both certified materials. The analyses of both water and plant materials show excellent repeatability (i.e. within-batch) with RSD (%) below 1.2%, and reproducibility (between-batch precision) below 9%, even for the digested INCT-PVTL-6 certified material (5% RSD). Note that the between-batch RSD (%) values for the water SRM represent the instrumental reproducibility, while for the plant material, it represents the overall reproducibility of the analytical procedure, including the ashing and acid digestion stages.

**Table 1:** Validation of the trace elemental analysis by ICP-MS using standard reference materials (SRM) NIST SRM1640a—Trace Elements in Natural Water and INCT-PVTL-6 (Polish Virginia Tobacco Leaves, Institute of Nuclear Chemistry and Technology)

(SRM) 1640a—Trace Elements in Natural Water					
Element	Certified value(μg/L)^a^	Repeatability (*n* = 6)		Reproducibility (*n* = 3)	
		Calculated value^b^ (μg/L)	RSD (%)^c^	Calculated value^b^ (μg/L)	RSD (%)
Al	52.6 ± 1.8	54.4 ± 0.5	0.9	53.8 ± 3.5	6.5
V	14.93 ± 0.21	14.0 ± 0.1	0.4	14.1 ± 0.2	1.4
Cr	40.22 ± 0.28	37.9 ± 0.1	0.3	38.3 ± 0.7	1.8
Mn	40.07 ± 0.35	38.6 ± 0.3	0.7	36.8 ± 1.6	4.3
Fe	36.5 ± 1.7	37.1 ± 0.2	0.5	36.5 ± 0.9	2.3
Ni	25.12 ± 0.12	23.9 ± 0.1	0.4	24.5 ± 0.6	2.4
Co	20.08 ± 0.24	19.1 ± 0.1	0.4	19.3 ± 1.0	5.2
Cu	85.07 ± 0.48	85.1 ± 0.8	1.0	85.0 ± 0.3	0.3
Zn	55.20 ± 0.32	53.8 ± 0.3	0.5	54.6 ± 4.9	9.0
As	8.010 ± 0.067	7.44 ± 0.03	0.4	7.40 ± 0.20	2.7
Se	19.97 ± 0.16	18.6 ± 0.1	0.5	19.6 ± 1.0	5.2
Mo	45.24 ± 0.59	43.0 ± 0.3	0.7	43.5 ± 1.1	2.4
Cd	3.961 ± 0.072	3.99 ± 0.04	1.0	3.86 ± 0.24	6.2
Sb	5.064 ± 0.045	5.05 ± 0.06	1.2	5.38 ± 0.33	6.1
Pb	12.005 ± 0.040	11.2 ± 0.1	1.1	10.7 ± 0.4	4.2
U	25.15 ± 0.26	25.4 ± 0.3	1.0	24.0 ± 1.3	5.3
INCT-PVTL-6 (Polish Virginia Tobacco Leaves)					
Element	Certified value^b^ (μg/kg d.w.)	Calculated value^b^ (μg/kg d.w.)	RSD (%)	Calculated value^b^ (μg/kg d.w.)	RSD (%)
Mo	396 ± 29	375 ± 4	1.0	381 ± 16	4.3

*RSD* relative standard deviation

^a^Mean ± 2*σ*, where *σ* is the SE

^b^Mean ± SD

With regards to the accuracy levels for the water analysis, good agreement was observed between the measured and certified values for most elements (Table 1). Application of a two-tailed Student *t* test for the comparison of the mean experimental values from the reproducibility data set (*n*=3) with the certified values at a 95% confidence level (*t*_crit_ = 4.30) shows only significant differences in the determination of V, Cr, As and Pb in the water SRM. However, it must be pointed out that the recovery values for these four elements still achieved satisfactory values between 90% and 94%.

In terms of the determination of Mo in the tobacco leaves reference material, a good recovery was obtained of approximately 96%, with no significant difference in the calculated value when compared to the certified value at the 95% confidence level.

## Results

### Hydroponic experiments using control tap water: effect of pH and Mo concentration on plant growth and Mo uptake

**Fig. 1:**
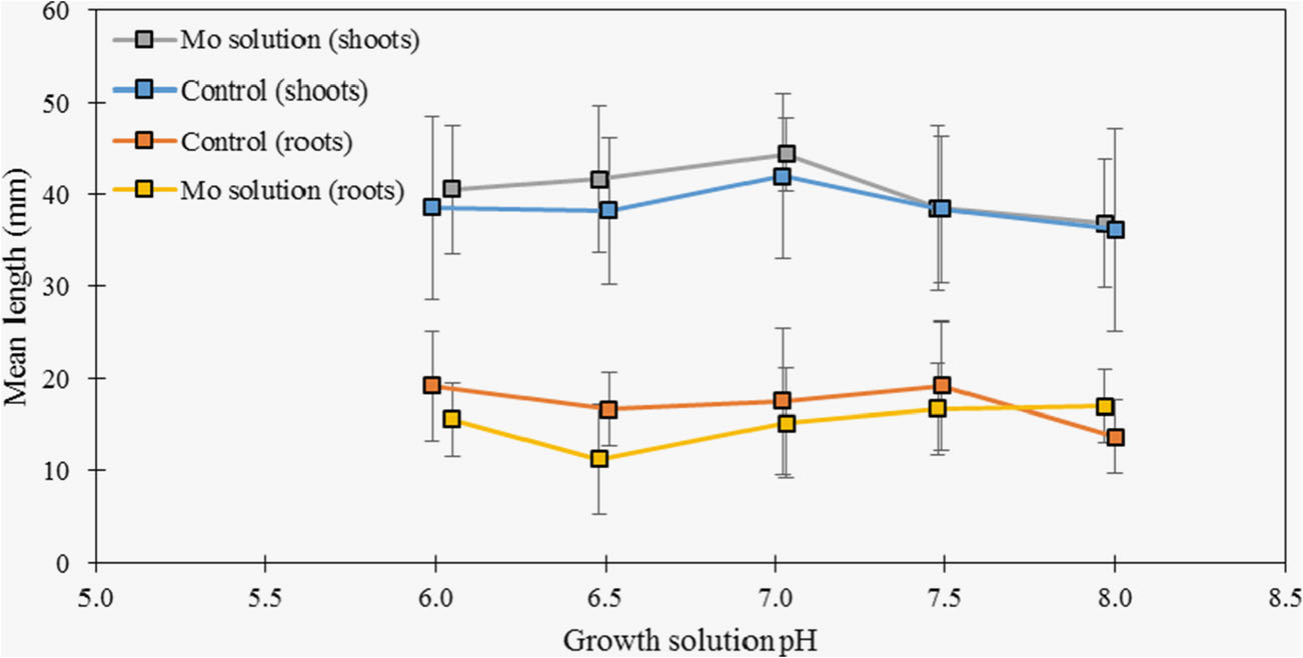
Shoot and root lengths of cress grown in control solutions (tap water, <0.1 μg/L Mo) and tap water solutions spiked with 150 μg/L Mo at varying pH (6.0–8.0). Mean ± SD (*n*=15)

A series of hydroponic experiments were undertaken to assess the effect of plant growth and Mo uptake by cress plants grown using control (no Mo addition) and Mo-spiked (150 μg/L) tap water solutions (with differing pH levels over the range 6.0 to 8.0). A slight increase in the uptake of Mo was confirmed as the pH rose to alkaline conditions (Table 2) for both the control and Mo-spiked tap water. Despite the raised levels of Mo in the plants grown in the spiked solutions, no reduction in plant growth was observed either for the roots or shoots, and no significant differences were found when errors were considered (see Fig. 1, mean ± SD for *n*=15).

**Table 2:** Molybdenum concentrations of cress samples grown at different pH levels (6.0–8.0) in control (tap water, TW) and 150 μg/L Mo-spiked growth solutions from hydroponic studies, mean ± SD (*n* = 2)

pH	Mo concentration of cress (mg/kg, d.w.)	
	Control (TW)	Molybdenum solution (150 μg/L Mo)
6.0	1.38 ± 0.49	31.6 ± 1.0
6.5	1.42 ± 0.08	33.0 ± 1.6
7.0	1.29 ± 0.22	37.5 ± 7.8
7.5	1.18 ± 0.14	36.6 ± 3.5
8.0	1.81 ± 0.76	36.4 ± 0.8

**Fig. 2:**
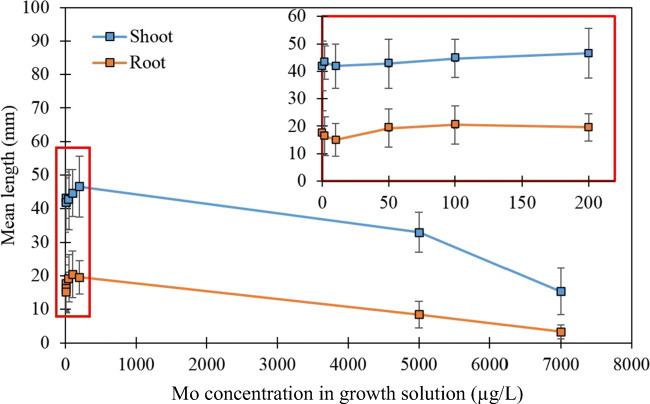
Shoot and root lengths of cress grown in tap water solutions at pH 7 with varying Mo dose (<0.1–7000 μg/L Mo). Mean ± SD. *Note:* the insert shows the relationship for cress grown in <0.1–200 μg/L Mo

**Fig. 3:**
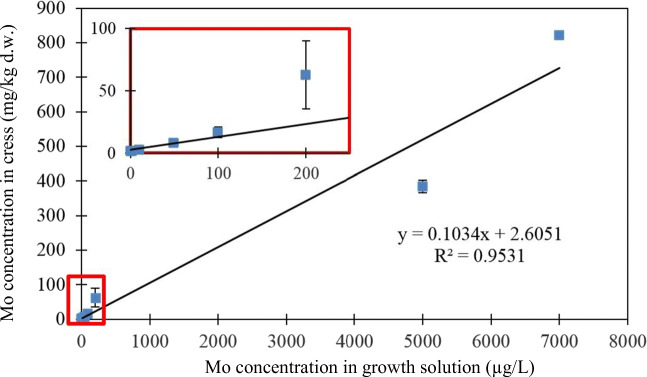
Mo concentrations in cress growing in hydroponic solutions with different Mo doses (<0.1–7000 μg/L Mo). Mean ± SD (*n*=2). *Note*: the insert shows the relationship for cress grown in <0.1–200 μg/L Mo

The effect of Mo dose on the growth of cress plants was investigated by increasing the Mo-spiked levels in the tap water from <0.1 (LOD) to 7000 μg/L Mo at pH 7. Although the maximum Mo-spiked level used in these experiments is higher than that reported by Jaafar (2018) for groundwater samples from La Pampa, it was set to investigate whether any Mo toxicity symptoms would be exhibited under hydroponic conditions. Figure 2 shows that there was little effect on plant growth at the lower concentrations of spiked Mo (<0.1–200 μg/L Mo in tap water). However, toxicity symptoms were observed in cress grown in the 5000 and 7000 μg/L Mo-spiked solutions. This was in the form of stunted roots and reduced plant growth (Fig. 2 and Fig. S1 in Supplementary Information). Analysis of the plant material by ICP-MS showed that Mo levels in the cress increased from 1.29 to 821 mg/kg Mo (d.w.) in the investigated range. Evaluation of the Pearson correlation of the data showed a statistically significant positive correlation (Fig. 3) between the Mo levels of the growth solution and that of the cress samples (*r* = 0.9531, a two-tailed Student *t* test was used to determine the level of significance of the regression coefficient, *t*_calc_= 11.04 > *t*_crit_ = 2.45, 95% confidence, based on 6 degrees of freedom, d.o.f.).

### Hydroponic experiments using tap water and groundwater from Eduardo Castex (La Pampa): effect of concomitant elements on Mo uptake

Groundwater samples collected and analysed from La Pampa (Argentina) have been reported to not only contain raised levels of Mo but also other elements (As, V, Fe, Mn) primarily arising from rhyolitic ash present in the loess deposits (Smedley and Nicolli 2014). These concomitant elements found in groundwater from La Pampa may influence the uptake of Mo by pasture plants grown on farms in this region. Moreover, the hydroponic experiments so far have only assessed the uptake of Mo in ‘ideal’ solutions. Groundwaters also contain other ‘nutrients’ (arising from loess deposits or soil layers) which will influence both the growth of the plant and the uptake of Mo. Therefore, a series of hydroponic experiments were undertaken to evaluate the capacity of plants to uptake Mo using cress (*L. sativum*) as the model cultivar, namely (1) synthetic solutions using tap water containing a Mo-spike of 150 μg/L and known levels of concomitant ions (Fe, Mn, Al) over the range of 20 to 1300 μg/L, based on previous multielement data of groundwater from La Pampa (O’Reilly et al. 2010; Watts et al. 2010; Farnfield 2012; Jaafar 2018); (2) commercial NKP plant feed solutions spiked with 150 μg/L Mo; and (3) two pooled groundwater samples (GW1 and GW2) collected from farm well near Eduardo Castex (La Pampa, Argentina) containing concentrations of Mo between 65.0 and 92.5 μg/L.

#### Effect of Fe, Mn and Al on Mo uptake

The justification for examining the effect of concomitant ions in these hydroponic experiments is based on publications reporting evidence of the adsorption of Mo, as MoO_4_^2−^, being influenced by Mn/Fe/Al oxides in soils, which is especially prevalent at more acidic pH levels (Kaiser et al*.* 2005). Moreover, it is known that in solution, easily mobile anions (such as MoO_4_^2−^) are readily co-precipitated by cations, including Mn^2+^ (Kabata-Pendias 2010). Therefore, to assess the hypothesis whether the presence of cationic species of Fe, Mn and Al would reduce the uptake of Mo under hydroponic conditions, a series of experiments were conducted where cress was grown in tap water containing 20–1000 μg/L Fe^2+^/Fe^3+^, 20–1300 μg/L Mn^2+^ and 10–500 μg/L Al^3+^ at pH 7 with 150 μg/L of Mo added as MoO_4_^2−^.

Table 3 shows the concentrations of Mo, Fe, Mn and Al in cress plants grown tap water spiked with these elements. The analyses of the plant material showed Mo levels ranging between 22.2 and 35.8 mg/kg (d.w.). Although at the highest level of the concomitant cations in solution, the concentrations of Fe, Mn and Al in the cress increased between 2- and 25-fold, when uncertainties were considered, no significant effect on the plant uptake of Mo was observed compared against the control experiment with 150 μg/L of Mo but in the absence of the concomitant ions.

**Table 3:** Elemental concentrations in cress samples grown in tap water spiked with 150 μg/L Mo, adjusted at pH 7, with and without the addition of concomitant ions: Fe (20–1000 μg/L), Mn (20–1300 μg/L) and Al (10–500 μg/L)

Concentration of concomitantions in spiked tap water (μg/L)*	Element concentration in cress (mean ± SD, mg/kg d.w., *n* = 2)			
	Mo	Fe	Mn	Al
No added concomitants	35 8 ± 3.0	69.8 ± 2.4	45.4 ± 1.1	4.70 ± 1.18
20 μg/L Fe	22.1 ± 2.6	**72.6 ± 2.5**	50.0 ± 1.1	5.30 ± 1.28
100 μg/L Fe	28.8 ± 1.3	**65.6 ± 2.2**	44.3 ± 0.9	4.50 ± 1.09
1000 μg/L Fe	33.1 ± 1.6	**100 ± 3**	43.3 ± 0.7	5.59 ± 1.35
20 μg/L Mn	30.6 ± 1.4	71.4 ±2.4	**53.0 ± 1.3**	3.66 ± 0.92
400 μg/L Mn	28.7 ± 3.4	63.5 ± 2.2	**198 ± 5**	4.65 ± 1.16
1300 μg/L Mn	27.8 ± 3.3	75.4 ± 2.6	**590 ± 11**	2.63 ± 0.68
10 μg/L Al	29.0 ± 2.2	68.5 ± 2.3	49.7 ± 1.3	**5.26 ± 1.32**
50 μg/L Al	23.2 ± 2.8	68.3 ± 2.4	48.0 ± 0.8	**10.4 ± 1.2**
500 μg/L Al	32.1 ± 1.5	65.2 ± 2.2	46.7 ± 1.0	**79.3 ± 9.5**

Values in bold characters indicate those samples in which element concentrations in the growth solutions were spiked over the backgound level

*All solutions containing the same concentration of Mo (150 μg/L)

#### Effect of nutrients on Mo uptake

The next hydroponic experiments investigated the effect of a nutrient solution being added to the Mo-spiked cress plants to assess the impact of major nutrients that would normally be found in the soil solution of pasture plants grown in La Pampa following irrigation with local groundwaters. A commercial plant feed (Baby Bio) concentrate contains NPK (nitrogen/phosphorus/potassium, 10.6:1.9:1.4) fertiliser and Mn and Zn chelated EDTA. Studies have shown that EDTA, which is a strong complexing agent, can enhance the extraction of heavy metals and metalloids from soil and thus increase the bioavailability to plants (Liphadzi and Kirkham 2006). In the case where cress was grown hydroponically in 0.01% commercial plant feed, solutions were prepared in DDW and spiked with 150 μg/L Mo at pH 7 (see composition of the solutions in Table 4). As expected, an enhanced germination was observed and significant increased cress shoot lengths occurred (+12%) in the presence of the plant feed due to the additional nutrients (42 ± 7 mm plant shoots grown in Mo-spike vs. 56 ± 7 mm for Mo-spike + 0.01% nutrient solutions, *t*_exp_=5.48 > *t*_crit_=1.70, 95% confidence, one-tail Student’s *t* test, d.o.f. = 28). On the other hand, little variation in root length was observed between the two studies (22 ± 7 for control vs. 20 ± 5 mm for nutrients + Mo, *n*=15). Interestingly, when looking at the elemental levels in the plant samples (Table 4), it can be observed that as the concentration of Mn, Fe and Zn increased in the solution, the levels of these elements also increased in the hydroponically grown cress samples. Furthermore, the concentration of Mo reduced by 24%, going from 33.0 to 25.1 mg/kg (d.w.).

**Table 4:** Trace element levels of growth solutions (tap water, commercial plant nutrient solution and groundwater from La Pampa, Argentina) and levels in cress hydroponically grown in the respective solutions

Element	Trace element levels in growth solution (mean ± SD μg/L, *n* = 3)				Trace element levels in plant material (mean ± SD mg/kg, d.w., *n* = 2)			
	Tap water+ Mo	Plant feed^#^in DDW + Mo	La Pampa GW1	La Pampa GW2	Tap water+ Mo	Plant feed^#^ inDIW + Mo	La Pampa GW1	La Pampa GW2
Al	1.60 ± 0.12	1.70 ± 0.21	19.0 ± 0.7	133 ± 1	3.9 ± 1.1	5.5 ± 1.4	18.5 ±2.2	58.0 ± 7.0
V	0.14 ± 0.01	0.08 ± 0.01	432 ± 9	278 ± 5	0.03 ± 0.00	0.02 ± 0.01	3.48 ± 0.87	0.83 ±0.25
Cr	0.53 ± 0.01	0.03 ± 0.01	1.08 ± 0.02	0.11 ± 0.01	1.12 ± 0.08	1.09 ± 0.09	0.92 ± 0.07	6.52 ± 2.05
Mn	0.31 ± 0.02	43.9 ± 0.4	0.29 ± 0.12	235 ± 6	49.1 ± 9.2	66.3 ± 5.7	39.5 ± 7.4	77.3 ± 6.6
Fe	4.66 ± 0.06	163 ± 6	24.0 ± 0.1	108 ± 7	68.1 ± 2.3	81.1 ± 5.8	66.6 ± 2.3	223 ± 16
Ni	2.67 ± 0.02	0.61 ± 0.01	1.46 ± 0.01	1.54 ± 0.10	5.4 ± 1.7	2.92 ± 0.20	1.97 ± 0.10	3.76 ± 0.25
Co	0.05 ± 0.01	0.29 ± 0.01	0.39 ± 0.04	0.80 ± 0.08	0.12 ± 0.01	0.10 ± 0.01	0.10 ± 0.01	0.53 ± 0.02
Cu	227 ± 1	11.6 ± 0.1	4.94 ± 0.10	3.14 ± 0.12	33.8 ± 4.2	15.8 ± 2.6	6.84 ± 1.12	8.80 ± 1.10
Zn	26.4 ± 0.2	71.5 ± 0.3	5.60 ± 0.11	17.4 ± 1.9	84.5 ± 4.2	103 ± 2	59.2 ± 0.9	134 ± 2
As	0.17 ± 0.02	0.03 ± 0.01	969 ± 64	23.7 ± 0.2	0.05 ± 0.01	0.06 ± 0.03	4.73 ± 1.07	6.94 ± 1.57
Se	< 0.4*	0.20 ± 0.01	17.7 ± 0.1	6.30 ± 0.15	0.18 ± 0.01	0.18 ± 0.00	0.16 ± 0.01	0.32 ± 0.05
**Mo**	**150**^##^	**150**^##^	**65.0 ± 0.6**	**92.5 ± 2.4**	**33.0 ± 3.9**	**25.1 ± 1.2**	**1.89 ± 0.20**	**4.59 ± 0.48**
Cd	< 0.002*	0.01 ± 0.00	0.07 ± 0.01	0.17 ± 0.01	0.36 ± 0.04	0.31 ± 0.02	0.34 ± 0.01	0.71 ± 0.04
Sb	0.10 ± 0.01	< 0.02*	0.80 ± 0.03	0.20 ± 0.01	< 0.002**	< 0.002**	< 0.002**	< 0.002**
Pb	0.21 ± 0.01	< 0.04*	< 0.04*	< 0.04*	0.07 ± 0.01	0.06 ± 0.02	0.07 ± 0.02	0.17 ± 0.01
U	0.16 ± 0.01	< 0.0002*	94.0 ± 3.7	14.9 ± 0.2	0.02 ± 0.01	0.02 ± 0.01	0.95 ± 0.08	0.79 ± 0.05

Values in bold characters indicate those samples in which element concentrations in the growth solutions were spiked over the backgound level

DDW: doubly distilled water (18 mΩ/cm); GW: groundwater

^#^Commercial plant nutrient solution, Baby Bio (0.01% in DDW)

^##^As spiked

*Below instrumental LOD (limit of detection)

**Below LOD for the analytical method (including sample pre-treatment and dilution)

#### Mo uptake from groundwater samples from La Pampa

The pooled water samples from La Pampa were also used in the hydroponic experiments (Table 4). Scoping experiments were carried out based on the original pH levels of the groundwater. However, due to the high acidity levels (as low as pH 0.9), no germination was observed over the 12-day period (Fig. S2). Therefore, the pH of the water was adjusted to ~7 (using ammonium hydroxide) for subsequent experiments, which had been observed to produce optimal germination for the cress (Section 3.1). It also must be noted that, although at pH <4, Mo may be present in solution as Mo^3+^ (Ochs et al. 2011), whereas the dominant species at pH 7 is MoO_4_^2−^. Therefore, the La Pampa groundwater experiments were directly comparable with the hydroponic studies using Mo-spiked Surrey tap water. The elemental concentrations for the two pooled groundwater samples are reported in Table 4. Even after pH adjustment, the use of the groundwater resulted in stunted growth of the cress, and there was a significant reduction of the mean shoot length (28 ± 3 and 36 ± 6 mm for GW1 and GW2, respectively, *n*=15) in comparison with the control tap water solutions spiked with 150 μg/L Mo at pH 7 (42 ± 7 mm). However, the effect on the root length was not so clear as there was a greater degree of variability; the control experiment (18 ± 4 mm) and the groundwater from La Pampa (15 ± 5 and 29 ± 10 mm for GW1 and GW2, respectively *n*=15). When looking at the uptake of Mo by the cress plants (Table 4), it is remarkable that although the concentration of Mo in Argentinean waters was only about 50% of the concentration of Mo added in the synthetic solutions (65.0–92.5 μg/L Mo compared with 150 μg/L for the spiked Mo), the levels in the cress plants had a much larger reduction. That is, 1.89–4.59 mg/kg (d.w.) for the plants grown in the La Pampa groundwater, 33.0 ± 4.0 mg/kg (d.w.) for the spiked tap water and 25.1 ± 1.2 mg/kg (d.w.) for the plant feed.

## Discussion

### Hydroponic experiments using tap water; effect of pH and Mo concentration

The concentrations of molybdenum in the cress plants grown in the control solution (<0.1 μg/L Mo) ranged from 1.38 to 1.81 mg/kg (d.w.), which is within the typical levels (0.07–2.50 mg/kg, d.w.) reported for various plants by Pais and Benton Jones (1997). Tejada-Jiménez et al*.* (2013) also reported typical levels of Mo in plant leaves of 0.1–1.0 mg/kg (d.w.), with most >0.2 mg/kg (d.w.). In tap water solutions spiked with 150 μg/L Mo, a significant uptake of Mo by the cress was observed (31.6–36.4 mg/kg Mo, d.w.) over the tested pH range of 6 to 8. At these pH values, the dominant species of Mo has been reported to be MoO_4_^2−^, while at pH below 4, Mo^3+^ may be present in the solution (Ochs et al*.* 2011). Previous studies suggest that below pH 4.2 Mo is not bioavailable to plants (Han 2007). However, this was not possible to be tested in the present study as the experiments carried out at low pH (pH 2.45 and 3.10) using the groundwater from La Pampa (Argentina) showed that plant growth was impaired (Fig. S2).

The uptake of Mo (possibly as MoO_4_^2−^) follows a linear trend over the range of 0 to 7000 μg/L Mo added to the tap water (pH 7), with clear symptoms of toxicity appearing at higher Mo concentrations, i.e. a slight yellowing of the leaves (Fig. S1) and stunted growth (Fig. 2). These observations seem to match with the symptoms predicted by McGrath et al*.* (2010), who reported chlorosis and depressed growth as visible effects of Mo toxicity for higher plants.

### Hydroponic experiments using tap and ground waters: effect of concomitant elemental ions

The effect of concomitant ions on the uptake of Mo by plants was tested for cress grown in tap water and commercial nutrient plant feed solutions spiked with Mo, and in groundwater with high natural levels of Mo (Jaafar et al*.* 2018).

#### Effect of Fe, Mn and Al on Mo uptake

In the first set of experiments, tap water solutions were spiked with 150 μg/L of Mo and 20–1000 μg/L Fe^3+^; 20–1300 μg/L Mn^2+^ or 10–500 μg/L Al^3+^ at pH 7. At this stage, there was no information in the literature to support whether adsorption or complexation of Mo (possibly as MoO_4_^2−^) with Fe, Mn or Al occurs in solution, thus reducing Mo uptake. Although some studies have suggested that plants may preferentially uptake cations over anions (Larcher 2003; White 2012), the results for the hydroponic experiments did not show any significant effect of the three cations on the uptake of Mo, even at the higher concentrations of the concomitant ions tested, as it can be observed in Table 3.

Raising the Fe concentration of the growth solution caused an increase (*t*_exp_= *t*_crit_ = 1.70, one-tail Student *t* test, 95% confidence, d.o.f. = 28) in the average shoot length (from 42 ± 7 mm without any added Fe to 47 ± 9 mm with 1000 μg/L Fe, Fig. S3). This agrees with Smolik et al*.* (2013) who also observed this trend in radish sprouts. With regards the uptake of Fe and Mo by the cress (Table 3), the level of Fe in the plant material increased linearly with the concentration of Fe in solution, and excellent correlation was found (*r*=0.9603, *t*_exp_= 5.56 > *t*_crit_ = 3.18, two-tail Student *t* test, 95% confidence, d.o.f. = 3). However, the presence of Fe did not cause a significant effect on the uptake of Mo even at the highest concentration of Fe in solution (35.8 ± 3.0 mg/kg Mo without any added Fe vs. 33.1 ± 1.6 mg/kg Mo with 1000 μg/L Fe, Table 3, *t*_exp_= 1.17 < *t*_crit_ = 4.30, two-tail Student *t* test, 95% confidence, d.o.f. = 2). This contrasts with previous research showing that Fe helped overcome the phenotypes associated with excess Mo, and vice versa. Similarly, Berry and Reisenauer (1967) observed an Fe–Mo inter-relationship, where the presence of molybdate significantly increased the ability of the plants to absorb Fe in tomato plants, confirmed by more recent evidence of the mutual interaction on their homeostatic regulation in other cultivars such as cucumbers (Vigani et al. 2017). Most molybdoenzymes present in plants require Fe-containing redox groups (Bittner, 2014). However, the inter-relationship between Fe and Mo is not fully understood, and a decrease in plant-available molybdate may result from the acidification of the rhizosphere during Fe deficiency (Bittner 2014). The Fe levels found in the cress of this study (62.0–100.0 mg/kg, d.w.) were similar to the reported levels (18 to 1000 mg/kg, d.w.) found in fodder plants (including *Brassica*, clover, alfalfa) (Kabata-Pendias 2010).

With regards Mn, Anderson and Arnot (1953) and Nayyar et al*.* (1980) reported that Mn in soils had an antagonistic impact on Mo uptake in plants. However, conflicting evidence was observed by Mulder (1954) who was unable to duplicate this interaction for all plant species investigated. A more recent publication by Rietra et al. (2017) evaluated the existing evidence in the literature with regards the interaction of macro- and micronutrients and the effect on the yield of multiple agricultural crops, concluding that there are no significant antagonistic or synergistic effects between Mo and Mn. This seems to be supported by the evidence in the present study, which does not show any reduction of Mo uptake in the presence of increased Mn^2+^ levels in the growth solution (35.8 ± 3.0 mg/kg Mo without any added Mn vs. 27.8 ± 3.3 mg/kg Mo with 1300 μg/L Mn, Table 3, *t*_exp_= 2.54 < *t*_crit_ = 2.92, one-tail Student *t* test, 95% confidence, d.o.f. = 2). The addition of Mn^2+^ resulted in significantly higher concentrations in the cress (from 53.0 to 590 mg/kg d.w. for plants cultivated in 20 to 1300 μg/L Mn^2+^) with excellent linearity and a significant correlation between the levels of Mn in solution and in the cress (*r*=0.9995, *t*_exp_= 43.4 > *t*_crit_ = 3.18, two-tail Student *t* test, 95% confidence, d.o.f. = 3), in agreement with previous publications reporting that the Mn levels in plants should be directly proportional to the concentration in the growth medium (Kabata-Pendias 2010; Ward 2000). The addition of Mn also caused a significant increase in the average root length (from 18 ± 4 mm without any added Mn to 26 ± 7 mm with 1300 μg/L Mn, Fig. S3, *t*_exp_ = 3.84 > *t*_crit_ = 1.70, one-tail Student *t* test, 95% confidence, d.o.f. = 28). This agrees with Waldren et al*.* (1987) who also reported the elongation of *Geum rival*e roots with an increasing Mn concentration of the growth solution. It is known that Mn, when in its soluble form (Mn^2+^), is rapidly taken up and translocated within plants and is typically found at levels of 20–240 mg/kg (d.w.) in plants. When the uptake of Mn^2+^ and Fe^3+^ are compared at highest levels, the data show that Mn is more effectively taken up by the cress since the presence of 1300 μg/L Mn^2+^ in solution resulted in a concentration of 590 mg/kg (d.w.) in the cress, while a similar concentration of 1000 μg/L Fe^3+^ only resulted in 100 mg/kg (d.w.) in the plants.

The addition of 10 to 500 μg/L Al^3+^ resulted in increased levels of Al in the cress, from 5.26 to 79.3 mg/kg (d.w.), showing excellent linear correlation between the level of Al in the plants and in solution in the whole tested range from 10 to 500 μg/L Al (*r*=0.9995, *t*_exp_= 43.4 > *t*_crit_ = 3.18, two-tail Student *t* test, 95% confidence, d.o.f. = 3). Moreover, the addition of Al resulted in reduced average root length by 23%, from 18 ± 4 mm without any added Al to 14 ± 6 mm with 500 μg/L Al (Fig. S3, *t*_exp_ = 2.14 > *t*_crit_ = 1.70, one-tail Student *t* test, 95% confidence, d.o.f. = 28). This observation agrees with plant studies conducted by Godbold and Jentschke (1998) and Choudhury and Sharma (2014), as Al in plants is often associated with toxicity, with the inhibition of roots being a visible phenotype (Mossor-Pietraszewska 2001). Despite the increased Al uptake and reduction in root length, the presence of Al did not seem to have any significant effect on the uptake of Mo, even at the highest level tested of 500 μg/L Al (35.8 ± 3.0 mg/kg Mo without any added Mn vs. 32.1 ± 1.5 mg/kg Mo with 500 μg/L Al, Table 3, *t*_exp_= 1.56 < *t*_crit_ = 4.30, two-tail Student *t* test, 95% confidence, d.o.f. = 2).

#### Effect of nutrients on Mo uptake

The uptake of Mn, Fe and Al by cress grown using a commercial plant nutrient feed was very similar to the values in the previous experiments using synthetic solutions (Section 4.2.1) and fitted well in the regressions between the concentrations of concomitant ions in solution and in the cress (regression coefficients going from *r* = 0.9693, 0.9995 and 0.9998 for spiked tap water data only to *r* = 0.9322, 0.9995 and 0.9992 including the data from the nutrients solution, for Fe, Mn and Al, respectively). This could suggest that the presence of the EDTA complexing agent in the commercial plant supplement does not enhance the bioavailability of these elements to plants. However, the levels of Mo in the cress grown in the plant feed were lower (25.1 ± 1.2 mg/kg Mo) than for the spiked tap water (33.0 ± 3.9 mg/kg Mo) despite having the same concentration of Mo in solution. Mn, Fe and Zn were the only elements that were present at higher concentrations in the plant feed than in the tap water spiked only with Mo (Table 4). In the previous set of experiments (Section 4.2.1), the presence of Fe or Mn (up to concentrations of 1000 and 1300 μg/L, respectively) did not have any significant effect on the uptake of Mo; therefore, it would be possible to attribute the reduced uptake of Mo to the presence of Zn in the commercial plant nutrient solution (71.5 vs. 26.4 μg/L Zn, Table 4). However, it must be noted that this slight difference is not statistically significant (*t*_exp_= 2.58 < *t*_crit_ = 4.30, two-tail Student *t* test, 95% confidence, d.o.f. = 2) and there is little information reported in the literature on any zinc–molybdenum relationship and therefore this could be a subject of further work.

#### Mo uptake from groundwater samples from La Pampa

The concentration of Mo in the pooled groundwater from La Pampa (Argentina) used for the hydroponic experiments (Table 4) presented values close to or over the former WHO guideline value of 70 μg/L for drinking water (WHO 2011). Furthermore, the Mo levels in groundwater exceeded the acceptable levels of the FAO (Food and Agriculture Organization) for irrigation water limits (10 μg/L Mo; FAO 1994). These concentrations are similar to O’Reilly (2010), who also found particularly high Mo levels in rural and urban wells in Eduardo Castex, La Pampa (15.3–1037 μg/L and 98.8–148 μg/L Mo, respectively), as well as Al-Rawahi (2016) in groundwater in various regions of southeast and central La Pampa, Argentina (0.2–1251 μg/L Mo). Elevated Mo levels present in these groundwaters primarily arise from the rhyolitic ash present in loess deposits (Grigg 1960; Smedley and Nicolli 2014).

In evaluating the plant uptake of Mo using groundwater from La Pampa, it is necessary to consider what the impact of other elements/ions present at naturally high level will be, beyond that for Fe, Mn and Al that have been studied in Section 4.2.1. Volcanic activity has had a major impact on the composition of sediments in La Pampa with frequent ash falls during the Tertiary and Quaternary Periods (Smedley et al. 2002). This is also the case for V, As, Se and U, which are commonly associated with naturally occurring Mo in rocks (Murcott 2012). In these groundwater samples, As, Se and U exceeded the respective WHO guideline values for drinking water (10 μg/L As; 10 μg/L Se; 15 μg/L U), while the other trace elements were found to be below the recommended limits, where available (WHO 2011). Levels of As in the measured groundwater were also in agreement with those reported by O’Reilly (2010) and Farnfield (2012), who determined levels in groundwater from Eduardo Castex of 3–1387 μg/L As and 24.9–777 μg/L As, respectively. WHO and FAO do not provide guidelines for V in drinking or irrigation water. However, FAO has established a maximum concentration of V in water destined for the watering of livestock of 100 μg/L (FAO 1994), and the Office of Environmental Health Hazard Assessment proposed a guideline of 15 μg/L V for drinking water (EHHA 2000). The V levels in pooled La Pampa groundwater were found to be above these limits (Table 4).

The average shoot length of cress grown in the two pooled Argentinean groundwaters (GW1 and GW2) was significantly shorter (28 ± 3 mm and 36 ± 6 mm) than that of cress grown in the Mo-spiked tap water (42 ± 7 mm). The reduction in the shoot length was statistically significant for both groundwater samples, compared with the spiked tap water (*t*_exp_ = 7.12 for GW1 and *t*_exp_ = 2.52 for GW2, > *t*_crit_ = 1.70, one-tail Student *t* test, 95% confidence, d.o.f. = 28) despite having comparable levels of Mo (150 μg/L Mo in the spiked tap water vs. 65.0 and 92.5 μg/L Mo for GW1 and GW2, respectively). Further evaluation of the data showed that the average lengths of shoot and root of cress grown in GW1 water (28 ± 3 and 15 ± 5 mm for shoots and roots, respectively) were shorter than for GW2 (36 ± 6 and 29 ± 10 mm, for shoots and roots, respectively) and these reductions in GW1 were statistically significant (*t*_exp_ = 4.60 for shoots and *t*_exp_ = 4.85 for roots, >*t*_crit_ = 1.70, one-tail Student *t* test, 95% confidence, d.o.f. = 28). Note that GW1 corresponded to the highest levels of As and V observed in the pooled groundwater (969 μg/L As and 432 μg/L V, Table 4). Excessive uptake of As by plants is toxic to them, due to competition and replacement of phosphate (a macronutrient required for plant growth) in enzymatic reactions, and toxicity symptoms include the inhibition of root growth (Meharg and Hartley-Whitaker 2002).

It should be noted that Mo, V, As, Sb and Se form oxyanions in water at neutral pH (Mitra 2015; Yanga et al. 2015). Some of these trace elements, including Se and V, are important factors in biological processes, while others including As and Sb are often toxic (Tchounwou et al*.* 2012). The similar water chemistry of these trace elements may result in potential competition with Mo, thus reducing its uptake by plants (Yanga et al. 2015). The Argentinean groundwater with the highest Mo concentration 92 μg/L (GW2) resulted in increased cress Mo levels that are above the typical range reported by Pais and Benton Jones (1997) in plants (0.7–2.5 mg/kg Mo d.w.). However, these levels were significantly lower (1.89 and 4.59 mg/kg Mo d.w. in GW1 and GW2, respectively) than would have been expected using an equivalent Mo concentration in the synthetic solutions. Levels of Mo of 8.2 and 16.6 mg/kg (d.w.) were measured in cress grown in synthetic solutions containing 50 and 100 μg/L Mo, respectively (Section 3.1 and Fig. 3). It is evident that the impact of the water chemistry and the presence of other trace elements or ions in the groundwater from La Pampa (Argentina) had an influence on the uptake of Mo by the cress. Furthermore, the difference in Mo levels in cress cannot be attributed just to a pH effect since all solutions were buffered to pH 7.

The levels of As in the two Argentinean waters were very different (969 and 23.7 μg/L As for GW1 and GW2, respectively), but this did not translate into significant differences in the As levels of the cress plants (4.73 ± 1.07 and 6.94 ± 1.57 mg/kg As in cress grown in GW1 and GW2, respectively; *t*_exp_ = 1.64 < *t*_crit_ = 4.30, two-tail Student *t* test, 95% confidence, d.o.f. = 2). McCarty et al. (2011) also reported low levels of As (<3.6 mg/kg d.w.) in plants grown in soils containing both high and low levels of arsenic. However, the As levels in the cress were nevertheless above the reported levels in plants grown in uncontaminated soils (0.009–1.5 mg/kg d.w.; Ward 2000).

Increased concentrations of V in the groundwater growth solutions resulted in raised levels in the cress (Table 4). Vachirapatama et al*.* (2011) observed a direct correlation between V levels found in water and plants. Jaafar (2018) also observed a significant Pearson correlation in pasture plants collected from Eduardo Castex, Argentina (*r* = 0.89, *p* ≤ 0.01). Only one of the cress samples, grown in the GW1 solution, was above (3.48 ± 0.87 mg/kg V) the reported typical levels of V found in plants (0.1–2.5 mg/kg d.w.; Ward 2000). As stated previously, low levels of Mo were observed in cress grown in groundwater from La Pampa, lower than would be expected in the synthetic solutions having equivalent Mo levels. V may partially replace Mo in nitrogenase systems found in plants depending on the V and Mo bioavailability in the growth medium (Nicholas 1975; Kabata-Pendias 2010). Preferential uptake or a competing effect of V may thus occur, resulting in a decrease in the Mo levels of the cress plants.

**Fig. 4:**
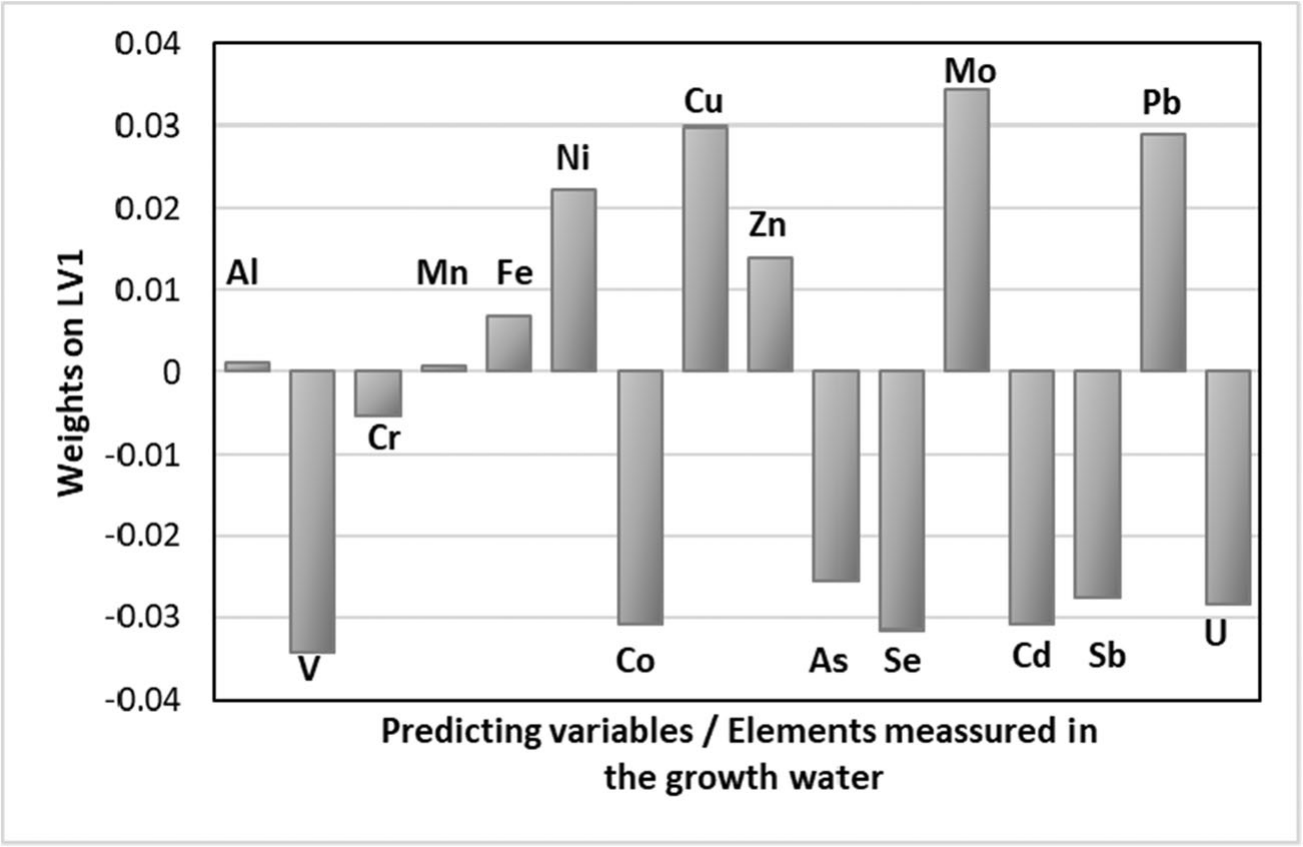
Representation of the weight vector (w_1_) of the first latent variable, obtained from the PLS model for the prediction of the levels of Mo in the cultivated cress using the concentration of elements present in the growth solutions as the predicting variables

Multivariate prediction models, in particular PLS (partial least squares), were applied to assess the interdependence between the composition of the water and the uptake of Mo by cress. In this study, the purpose of the PLS models was to determine the relative importance of the different ions in solutions (predictor variables, matrix X) on the concentration of Mo in the plants (predicted variable, vector y). The aim was not to develop a good predictive model, but to establish what trace elements have a stronger impact on the observed concentration of Mo in the cress, and from it to infer whether there are synergic or competition effects. The models were constructed using the concentrations of the 16 ions analysed in the growth solutions (see Table 4 for list) as predicting variables (X, 13×16). Both X and y blocks were autoscaled (mean centred followed by normalisation by the SD of each variable) to eliminate scale bias and models were validated using the leave-one-out algorithm. PLS models produce latent variables (LVs) that are linear combinations of the original predictor X variables, covarying optimally with the sought predicted values in y. PLS models search for the latent variables that maximise the covariance between X (concentration of trace elements in the growth solutions) and y (Mo in the cress) blocks. In this case, a 3-LV PLS model explained 81.2 and 89.6% of the variance of X and y, respectively, of which 53.0 and 85.2 corresponded to the first LV, also respectively. Therefore, the discussion of the relative importance of the concentration of the ions in solution in terms of the accumulation of Mo could be made based on the weight vector for the first score or latent variable, w_1_. The values are shown in Fig. 4. Direct comparisons can be done because of the autoscaling of both y and X. The larger the weight in absolute value for one particular X variable, the higher the effect of it on the explanation on the correlation with the y variable, i.e. the concentration of Mo in the grown plants. The values of w_1_ in Fig. 4 show the factor that has a higher impact on the concentration of Mo in the cress is the level of Mo in solution, followed by V with a similar weight in absolute value but with a negative effect. Other elements in solution show a negative impact on Mo uptake (Cr, Co, As, Se, Cd, Sb and U), which suggests that there is a preferential uptake or competing effect of these elements. Note that in the pooled Argentinean groundwater samples (Table 4), the highest concentrations in solution were those of As and V. Other trace elements like Al and Mn seemed to have little effect on the uptake of Mo, either positive or negative.

## Conclusions

A significantly positive Pearson correlation was observed (*r* = 0.9531, *p* ≤ 0.05) between Mo concentrations in the cress (1.29–821 mg/kg d.w.) and the hydroponic growth solutions (0–7000 μg/L Mo). Toxicity symptoms, in the form of stunted root growth, were exhibited at the highest Mo concentrations (>5000 μg/L Mo), with a few leaves demonstrating chlorosis at 7000 μg/L. An investigation using pooled groundwater samples from La Pampa (Argentina) as growth solutions (65.0–92.5 μg/L Mo) resulted in significantly lower cress Mo levels (1.89–4.59 mg/kg d.w.) than would have been expected using an equivalent Mo concentration in synthetic tap water solutions. Multivariate statistical evaluation of the concentration of ions in solution and the Mo in the cress suggests that there is preferential uptake or competition of other trace elements or ions in the Argentinean groundwater, including V and As. This study provides valuable data on the potential effect of using groundwater in La Pampa and other regions of Argentina for the irrigation of pastures. The naturally high levels of As, V, Mo, Fe, Mn and possibly U due to the geochemistry of the Quaternary loess deposits in the Chaco-Pampean Plain (Argentina) will lead to enhanced uptake of these elements in the plants, which may pose a possible toxic effect to agricultural crops and grazing livestock. This is an area of research that needs further investigation.

## Supplementary Information

ESM 1 (DOCX 2232 kb)

## Data Availability

All data generated or analysed during this study are included in this published article and its supplementary information files.

## References

[CR1] Al-Rawahi WA (2016) Vanadium, arsenic and fluoride in natural waters from Argentina and possible impact on human health. PhD Thesis, University of Surrey

[CR2] Al-Rawahi WA, Ward NI (2017). Field-based application of developed solid phase extraction with inductively coupled plasma mass spectrometry for vanadium speciation analysis of groundwaters from Argentina. Talanta.

[CR3] Anderson AJ, Arnot RH (1953). Fertilizer studies on basaltic red loam soil from the Lismore District, New South Wales. Aust J Agric Resour Econ.

[CR4] Berry JA, Reisenauer HM (1967). The influence of molybdenum on iron nutrition of tomato. Plant Soil.

[CR5] Bittner F (2014). Molybdenum metabolism in plants and crosstalk to iron. Front Plant Sci.

[CR6] Broadley M, Brown P, Cakmak I, Rengel Z, Zhao F (2012) Function of nutrients: micronutrients. In: Marschner P (ed) Marschner's mineral nutrition of higher plants, 3rd edn. Academic Press, pp 191–248

[CR7] Choudhury S, Sharma P (2014). Aluminum stress inhibits root growth and alters physiological and metabolic responses in chickpea (*Cicer arietinum L.*). Plant Physiol Biochem.

[CR8] Davis RD, Beckett PHT, Wollan E (1978). Critical levels of twenty potentially toxic elements in young spring barley. Plant Soil.

[CR9] FAO (1994) Water quality for agriculture, water quality for livestock and poultry, No. 6. http://www.fao.org/docrep/003/T0234E/T0234E06.htm Accessed 24 May 2019

[CR10] Farnfield HR (2012) Arsenic (total and speciation) in water from Argentina and its impact on human health. PhD Thesis, University of Surrey

[CR11] Frisbie SH, Mitchell EJ, Sarkar B (2015). Urgent need to reevaluate the latest World Health Organization guidelines for toxic inorganic substances in drinking water. Environ Health.

[CR12] Godbold DL, Jentschke G (1998). Aluminium accumulation in root cell walls coincides with inhibition of root growth but not with inhibition of magnesium uptake in Norway spruce. Physiol Plant.

[CR13] Grigg JL (1960). The distribution of molybdenum in the soils of New Zealand. New Zeal J Agr Res.

[CR14] Han FX (2007) Biogeochemistry of trace elements in arid environments. Springer

[CR15] Jaafar M (2018) Trace elements in natural water: the impact on quality, food preparation and production. PhD Thesis, University of Surrey

[CR16] Jaafar M, Marcilla AL, Felipe-Sotelo M, Ward NI (2018) Effect of food preparation using naturally-contaminated groundwater from La Pampa, Argentina: estimation of elemental dietary intake from rice and drinking water. Food Chem 246:258–265 10.1016/j.foodchem.2017.11.019 29291847

[CR17] Kaiser BN, Gridley KL, Brady JN, Philips T, Tyerman SD (2005) The role of molybdenum in agricultural plant production. Ann Bot 96:745–754 10.1093/aob/mci226 PMC4247040 16033776

[CR18] Kaiser BN, Gridley KL, Brady JN, Philips T, Tyerman SD (2005) The role of molybdenum in agricultural plant production. Ann Bot 96:745–754 10.1093/aob/mci226 PMC4247040 16033776

[CR19] Larcher W (2003) Physiological plant ecology: ecophysiology and stress physiology of functional groups. Springer

[CR20] Liphadzi MS, Kirkham MB (2006). Availability and plant uptake of heavy metals in EDTA-assisted phytoremediation of soil and composted biosolids. S Afr J Bot.

[CR21] May TW, Wiedmeyer RH (1998). A table of polyatomic interferences in ICP-MS. At Spectrosc.

[CR22] McCarty KM, Hanh HT, Kim K (2011). Arsenic geochemistry and human health in South East Asia. Rev Environ Health.

[CR23] McGrath SP, Micó C, Curdy R, Zhao FJ (2010). Predicting molybdenum toxicity to higher plants: influence of soil properties. Environ Pollut.

[CR24] Meharg AA, Hartley-Whitaker J (2002). Arsenic uptake and metabolism in arsenic resistant and nonresistant plant species. New Phytol.

[CR25] Mitra GN (2015) Regulation of nutrient uptake by plants: a biochemical and molecular approach. Springer

[CR26] Mossor-Pietraszewska T (2001). Effect of aluminium on plant growth and metabolism. Acta Biochim Pol.

[CR27] Mulder EG (1954). Molybdenum in relation to growth of higher plants and micro-organisms. Plant Soil.

[CR28] Murcott S (2012) Arsenic contamination in the world. IWA Publishing

[CR29] Nayyar VK, Randhawa NS, Pasricha NS (1980) Effect of interaction between molybdenum and copper on the concentration of these nutrients in berseem and its yield. Indian J Agric Sci 50:434–440

[CR30] Nicholas D (1975) The functions of trace elements in plants. In: Nicholas DJD, Egan AH (eds) Trace elements in soil-plant-animal systems, 1st edn. Academic Press Inc, pp 181–198

[CR31] O’Reilly JE (2010) Arsenic speciation in environmental and biological samples from Argentina: relationship between natural and anthropogenic levels and human health status. PhD Thesis, University of Surrey

[CR32] Ochs M, Vielle-Petit L, Wang L Mallants D (2011) Additional sorption parameters for the cementitious barriers of a near-surface repository. NIROND–TR 2010–06 E V1.

[CR33] Office of Environmental Health Hazard Assessment (2000) Proposed action level for vanadium. Water Toxicology Unit, Pesticide and Environmental Toxicology Section, Division of Drinking Water and Environmental Management Branch, Department of Health Services, USA

[CR34] O'Reilly J, Watts MJ, Shaw RA, Marcilla AL, Ward NI (2010). Arsenic contamination of natural waters in San Juan and La Pampa, Argentina. Environ Geochem Hlth.

[CR35] Pais I, Benton Jones J Jr (1997) The handbook of trace elements. CRC Press LLC.

[CR36] Rietra RPJJ, Heinen M, Dimkpa CO, Bindraban PS (2017). Effects of nutrient antagonism and synergism on yield and fertilizer use efficiency. Commun Soil Sci Plant Anal.

[CR37] Smedley P, Nicolli H, Viswanathan SS, Lopatin SI (2014). Molybdenum distributions and controls in groundwater from the Pampean aquifer of La Pampa Province. Molybdenum and its compounds: applications, electrochemical properties and geological implications.

[CR38] Smedley PL, Nicolli HB, Macdonald DMJ, Barros AJ, Tullio JO (2002). Hydrogeochemistry of arsenic and other inorganic constituents in groundwaters from La Pampa, Argentina. Appl Geochem.

[CR39] Smolik B, Cichocka J, Materny A, Śnioszek M, Zakrzewska H (2013). Effect of iron deficiency and excess on biometric and biochemical parameters indicated in the radish sprouts (*Raphanus sativus L. Subvar. radicula pers*.). J Inst Environ Protect.

[CR40] Tchounwou PB, Yedjou CG, Patlolla AK, Sutton DJ (2012). Heavy metals toxicity and the environment. Exp Suppl.

[CR41] Tejada-Jiménez M, Chamizo-Ampudia A, Galván A, Fernández E, Llamas Á (2013). Molybdenum metabolism in plants. Metallomics.

[CR42] Vachirapatama N, Jirakiattikul Y, Dicinoski G, Townsend AT, Haddad PR (2011). Effect of vanadium on plant growth and its accumulation in plant tissues. J Sci Technol.

[CR43] Vigani G, Di Silvestre D, Agresta AM, Donnini S, Mauri P, Gehl C, Bittner F, Murgia I (2017). Molybdenum and iron mutually impact their homeostasis in cucumber (*Cucumis sativus*) plants. New Phytol.

[CR44] Waldren S, Davies MS, Etherington JR (1987). The effect of manganese on root extension of *Geum rivale L.*, *G. urbanum L.* and their hybrids. New Phytol.

[CR45] Ward NI (2000) Trace elements. In: Fifield FW, Haines PJ (eds) Environmental analytical chemistry, 2nd edn. Blackwell Science Ltd, pp 360–392

[CR46] Watts MJ, O'Reilly J, Marcilla AL, Shaw RA, Ward NI (2010). Field based speciation of arsenic in UK and Argentinean water samples. Environ Geochem Hlth.

[CR47] White PJ (2012) Ion uptake mechanism of individual cells and roots: short distance transport. In: Marschner P (ed) Marschner's mineral nutrition of higher plants, 3rd edn. Academic Press, pp 7–47

[CR48] WHO (2011) Guidelines for drinking-water quality. World Health Organisation. https://www.who.int/water_sanitation_health/publications/2011/dwq_guidelines/en/ Accessed 19 Sept 2019

[CR49] Yanga N, Welch KA, Mohajerin TJ, Telfeyan K, Chevis DA, Grimm DA, Lyons WB, White CD, Johannesson KH (2015). Comparison of arsenic and molybdenum geochemistry in meromictic lakes of the McMurdo Dry Valleys, Antarctica: implications for oxyanion-forming trace element behavior in permanently stratified lakes. Chem Geol.

