# Supplementary material for: Investigation of the uptake of molybdenum by plants from Argentinean groundwater
Source: Environ Sci Pollut Res Int. 2021 Apr 30;28(35):48929–41. doi: 10.1007/s11356-021-13902-w (PMC8410703; doi:10.1007/s11356-021-13902-w)
Supplement: Supplementary file 1 — (DOCX 2232 kb) [file 11356_2021_13902_MOESM1_ESM.docx]

Supplementary Information:

**Investigation of the uptake of molybdenum by plants from Argentinean groundwaters**

K. Lawson-Wood^a,^ *, M. B. Jaafar ^a, b^, M. Felipe-Sotelo^a, ǂ^, N. I. Ward^a^

^a^ ICP-MS Facility, Chemistry Department, University of Surrey, Guildford, Surrey GU2 7XH, United Kingdom.

^b^ Faculty of Science and Marine Environment, Universiti Malaysia Terengganu, 21030 Kuala Nerus, Terengganu, Malaysia
* Currently at Perkin Elmer, Chalfont Road, Seer Green, Buckinghamshire, HP9 2FX, UK

ǂ Corresponding author, [m.felipe-sotelo@surrey.ac.uk](mailto:m.felipe-sotelo@surrey.ac.uk), Tel. +44 1483 686837

**Table S1.** Operating parameters for the inductively coupled plasma mass spectrometer
(Agilent 7700x).

| **Stage** | **Parameter** | **Typical operating conditions** |
| --- | --- | --- |
| **Collision/ reaction cell** | ON | He mode |
|  | Helium gas flow rate (mL min^-1^) | 4.8 |
| **Analysis** | Analyte isotopes | ^27^Al, ^51^V, ^55^Mn, ^56^Fe, ^63^Cu, ^66^Zn, ^75^As, ^95^Mo |
|  | Internal standard element | ^115^In |
| **Detector  (electron multiplier)** | Pulse HV (V) | 980 |
|  | Analog HV (V) | 1680 |

(5) 200 µg/L Mo (6) 5000 µg/L Mo (7) 7000 µg/L Mo

(1) 2 µg/L Mo (2) 10 µg/L Mo (3) 50 µg/L Mo (4) 100 µg/L Mo

**Figure S1.** Cress grown in spiked tap water at pH 7.0 with increasing molybdenum dose
(2 – 7000 µg/L Mo). Images show germination after a 12-day period.


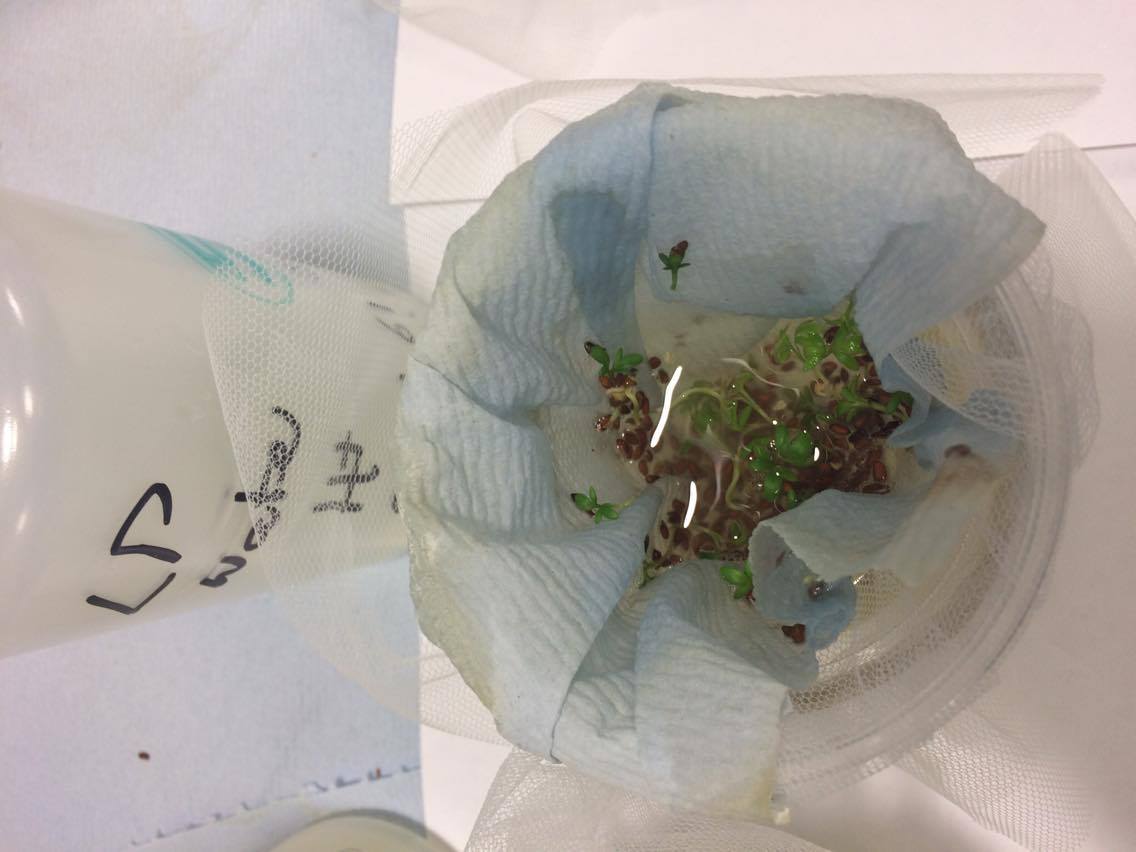

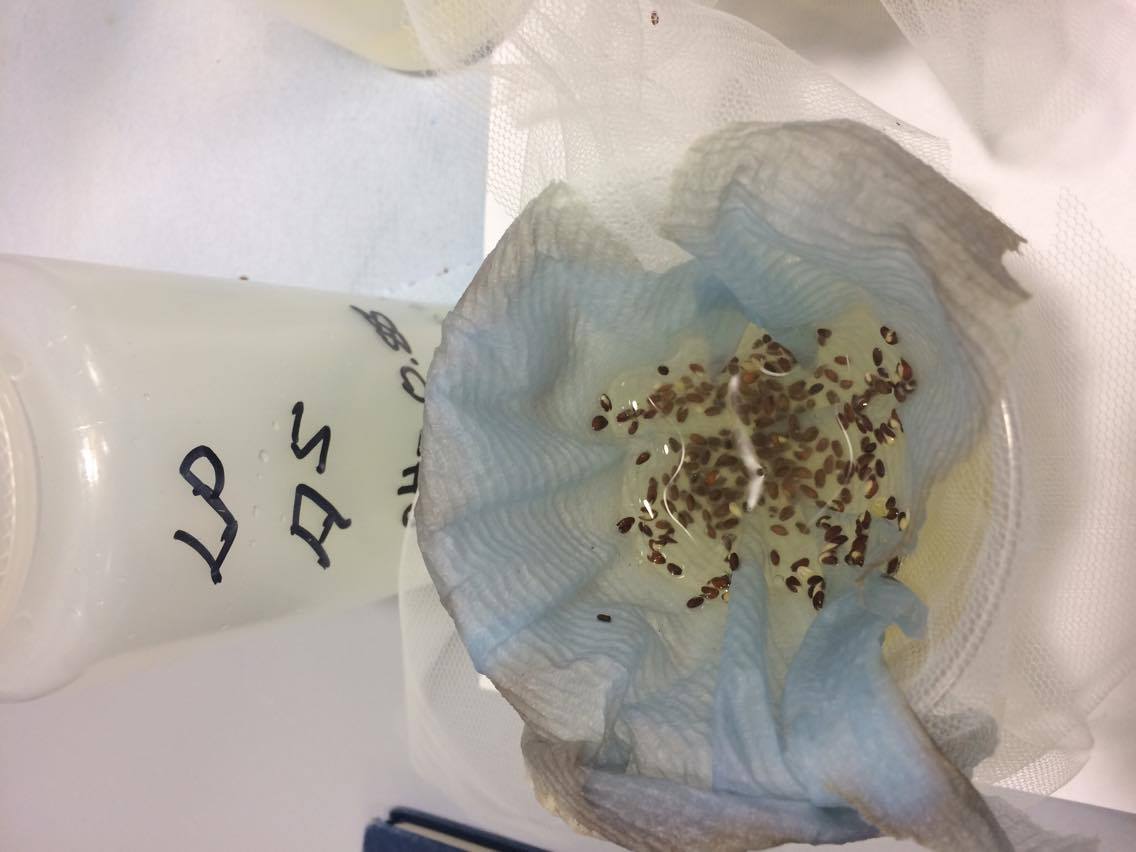

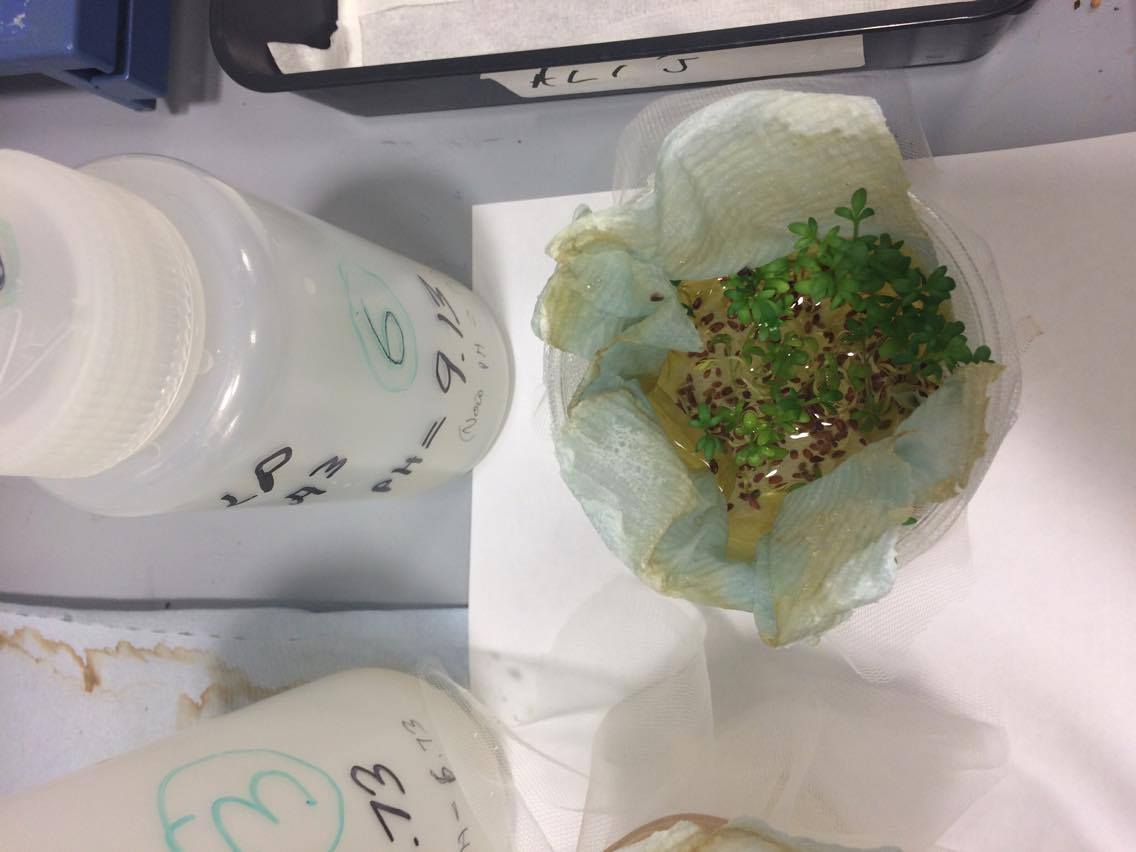

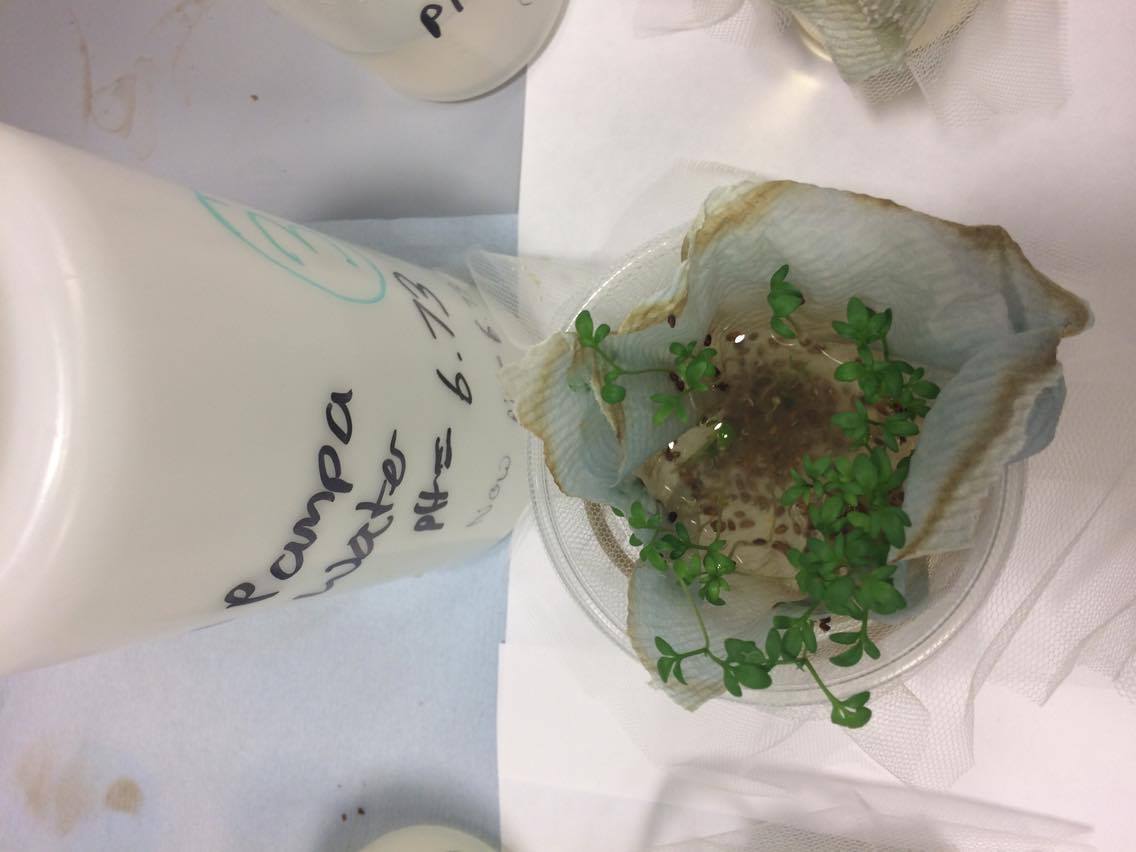


(1) LP-A La Pampa groundwater

(2) LP-B La Pampa groundwater

(3) GW1 La Pampa groundwater

(4) GW2 La Pampa groundwater

**Figure S2.** Hydroponic growth experiment using four pooled groundwaters from La Pampa, Argentina, at the original pH of the solutions (1 and 2) and at pH 7 (3 and 4). Images show germination after a 12-day period.

**Figure S3.** Lengths of shoot and root (mean ± SD, n=15) of cress plants grown in tap water with varying added concentrations of Fe (20 – 1000 µg/L), Mn (20 - 1300 µg/L) and Al (10 – 500 µg/L). All solutions were adjusted to a pH of 7 and spiked with 150 µg/L Mo
